# Acoustic Emission-Based Pipeline Leak Detection and Size Identification Using a Customized One-Dimensional DenseNet

**DOI:** 10.3390/s25041112

**Published:** 2025-02-12

**Authors:** Faisal Saleem, Zahoor Ahmad, Muhammad Farooq Siddique, Muhammad Umar, Jong-Myon Kim

**Affiliations:** 1 Department of Electrical, Electronics and Computer Engineering, University of Ulsan, Ulsan 44610, Republic of Korea; faisal1999@mail.ulsan.ac.kr (F.S.); zahooruou@mail.ulsan.ac.kr (Z.A.); mfarooq229@mail.ulsan.ac.kr (M.F.S.); muhammadumar@mail.ulsan.ac.kr (M.U.); 2 PD Technology Co., Ltd., Ulsan 44610, Republic of Korea

**Keywords:** acoustic emission, pipeline leakage, DenseNet, one-dimensional, empirical wavelet transform, deep learning

## Abstract

Effective leak detection and leak size identification are essential for maintaining the operational safety, integrity, and longevity of industrial pipelines. Traditional methods often suffer from high noise sensitivity, limited adaptability to non-stationary signals, and excessive computational costs, which limits their feasibility for real-time monitoring applications. This study presents a novel acoustic emission (AE)-based pipeline monitoring approach, integrating Empirical Wavelet Transform (EWT) for adaptive frequency decomposition with customized one-dimensional DenseNet architecture to achieve precise leak detection and size classification. The methodology begins with EWT-based signal segmentation, which isolates meaningful frequency bands to enhance leak-related feature extraction. To further improve signal quality, adaptive thresholding and denoising techniques are applied, filtering out low-amplitude noise while preserving critical diagnostic information. The denoised signals are processed using a DenseNet-based deep learning model, which combines convolutional layers and densely connected feature propagation to extract fine-grained temporal dependencies, ensuring the accurate classification of leak presence and severity. Experimental validation was conducted on real-world AE data collected under controlled leak and non-leak conditions at varying pressure levels. The proposed model achieved an exceptional leak detection accuracy of 99.76%, demonstrating its ability to reliably differentiate between normal operation and multiple leak severities. This method effectively reduces computational costs while maintaining robust performance across diverse operating environments.

## 1. Introduction

Pipelines are an essential backbone of modern infrastructure, enabling the cost-effective and efficient transportation of essential resources such as gas, water, and oil over long distances [1]. They offer a reliable and economical means of resource delivery, making them a key component in all industries [2,3]. However, despite their critical role, pipelines are vulnerable to various forms of degradation over time [4,5]. Corrosion, fatigue cracks, material defects, and external factors like natural disasters can all lead to significant wear and tear [6]. These factors can result in leaks, cracks, or even catastrophic ruptures, all of which pose severe risks to public safety, contribute to environmental pollution, and lead to substantial economic losses [7,8,9]. For instance, notable incidents such as the petroleum pipeline explosion in Hidalgo, Mexico, which tragically caused 120 fatalities, and the diesel pipeline leak in Guizhou, China, which led to financial losses totaling RMB 1.5 million, serve as striking reminders of the potentially devastating consequences of undetected pipeline leaks [10,11].

To reduce these risks, the early diagnosis of pipeline leaks is important for preventing such disasters and minimizing the associated environmental and economic impacts [12,13]. Detecting leaks in their early stages enables timely maintenance, thereby preventing small issues from escalating into larger and more costly problems [14]. This is where non-destructive testing methods have gained importance as essential tools for evaluating pipeline integrity without disrupting operations [15]. Among these techniques, AE technology has emerged as one of the most promising due to its high sensitivity to real-time changes in structural integrity [16]. AE technology detects stress waves generated by damage, allowing continuous monitoring of pipelines without requiring service interruptions. This capability to capture early indications of damage makes AE invaluable in maintaining the long-term health of pipelines [17,18].

The integration of machine learning (ML) and artificial intelligence (AI) technologies has further enhanced the effectiveness of AE-based monitoring systems [19,20]. By utilizing advanced algorithms, such as convolutional neural networks (CNNs) and support vector machines (SVMs), AE data can be analyzed with enhanced precision, facilitating the detection of patterns that indicate potential leaks [21]. These AI-driven approaches help minimize false alarms and improve the accuracy of leak detection [22]. However, beyond identifying the presence of a leak, the ability to determine the size of the leak is critical for assessing the severity of the issue and guiding appropriate maintenance decisions. Small leaks might be addressed with localized repairs, while larger leaks could require more significant interventions [23]. Accurate leak size identification is thus important in ensuring efficient, cost-effective repairs, reducing unnecessary operational downtime, and preventing further damage to the pipeline [24].

### Related Research Work

AE technology has become essential for diagnosing pipeline systems, especially in detecting leaks and assessing overall pipeline integrity. When a leak occurs, elastic waves are generated, which produce AE events that are captured by AE sensors positioned along the pipeline, resulting in AE hits [25]. The variations in these AE signals are instrumental in identifying leaks, as AE sensors detect stress waves emitted from structural changes within the pipeline [26]. Researchers have extensively focused on using these AE signal variations through feature extraction and pattern recognition methods across frequency domain, time domain, and time-frequency domain [27].

In time domain analysis, AE signals are examined based on temporal variations in signal characteristics, though pre-processing is often needed to minimize noise and interference [28]. Techniques like AE counts and cumulative AE energy have been used alongside machine learning algorithms, SVMs, and neural networks to detect leaks effectively [29]. Xu et al. [11] proposed a method for water pipeline leak detection by employing variational mode decomposition for noise reduction and Mel frequency cepstral coefficients for extracting essential features, achieving an accuracy of 93% for leak detection. However, while time domain methods are straightforward, they are sensitive to noise, which can affect accuracy in complex scenarios involving multiple interference sources. Frequency domain analysis offers a robust but complex alternative, focusing on the frequency spectrum of AE signals, making it valuable for detecting leak-specific amplitude and spectral characteristics in stationary signals [30,31]. Pipeline signals are often non-stationary, limiting the effectiveness of these methods. To address these non-stationary signals, time-frequency techniques like wavelet transforms and empirical mode decomposition have been applied; however, these methods have high computational costs [32]. Wang et al. [15] developed a hybrid leak detection system that integrated acoustic and pressure sensor data using dual Pearson threshold Ensemble Empirical Mode Decomposition (EMD) and one-dimensional CNN. By combining both sensor types after noise reduction, the method achieved improved accuracy, demonstrating the potential of hybrid approaches in analyzing non-stationary signals.

Increasingly, studies have explored hybrid approaches that integrate various feature extraction techniques with deep learning methods, such as CNN and autoencoders, to automate feature extraction and pattern recognition [33]. These approaches have achieved high precision in leak detection by utilizing techniques like short-time Fourier transform and continuous wavelet transform (CWT) for a more comprehensive analysis of temporal and spectral information [34]. For instance, combining CWT-generated scalograms with convolutional autoencoders has enhanced the extraction of global and local features, significantly improving leak detection accuracy across various pipeline conditions [35].

Recent advancements in fault detection methodologies have utilized deep learning, transfer learning, digital twin frameworks, and magnetic flux leakage (MFL) techniques to enhance predictive maintenance strategies. The integration of deep neural networks with transfer learning approaches [36] has demonstrated improved classification performance in industrial monitoring applications, aligning with the feature optimization strategies employed in this study. Additionally, digital twin-driven fault diagnosis models [37] offer significant advantages in simulating real-world fault conditions to enhance model generalization, an approach that could be extended to AE-based pipeline monitoring. In parallel, MFL detection techniques have been effectively applied for ferromagnetic pipeline weld inspections, providing insights into potential hybrid detection strategies [38]. These studies highlight the growing trend in multi-modal fault diagnosis, reinforcing the significance of adaptive learning and hybrid sensor fusion methodologies for improving real-time pipeline leak detection and size classification [39]. For natural gas pipelines, recent innovations, such as distributed feedback fiber laser vibration sensors combined with specialized neural network models, have shown promising results. These models integrate multi-scale convolutional neural networks with local feature extraction blocks, which process the original one-dimensional signal while preserving the time-domain correlation of leakage signals [40]. This approach effectively overcomes the challenges of feature preservation commonly associated with traditional signal-processing methods [41]. By utilizing improved complete ensemble EMD with adaptive noise and probabilistic neural networks (PNNs), the accuracy of pipeline leak detection has been enhanced. This process decomposes AE signals into intrinsic mode functions (IMFs), which are then used for time-frequency analysis to create feature vectors for training PNN models, resulting in more accurate leak detection [42]. Recent advancements have also emphasized integrating ground-penetrating radar (GPR) with deep learning and signal processing techniques like wavelet transform and ResNet-50, allowing detailed analysis of time-frequency characteristics in GPR data [43]. By transforming GPR bright scan images into time-frequency scale images and applying deep learning for feature extraction, researchers have achieved improved leak detection across various pipeline conditions [44]. Recent advancements in artificial intelligence have significantly improved fault diagnosis and prognosis, especially in scenarios with incomplete industrial data [45]. Studies have explored deep learning-based fault detection approaches, emphasizing the robustness of CNNs, probabilistic neural networks, and transfer learning techniques for predictive maintenance in complex industrial environments [46]. In particular, AI-driven fault prediction methods have been proposed to address challenges created by incomplete datasets, utilizing probabilistic models and uncertainty quantification techniques to enhance reliability and robustness [47]. Moreover, recent research has highlighted the importance of explainable AI in industrial applications, ensuring transparency and interpretability in automated decision-making processes [48].

Traditional time-domain techniques are highly sensitive to noise, significantly suppressing their reliability in real-world pipeline monitoring scenarios. Frequency-domain methods, although effective for stationary signals, face intrinsic limitations when dealing with the dynamic and non-stationary nature of pipeline signals, reducing their applicability in diverse operational conditions. Similarly, time-frequency techniques, while offering a more comprehensive representation of signals, come with trade-offs such as high computational costs and the need for precise parameter tuning, making them less feasible for large-scale, real-time implementations.

To overcome these challenges, this research introduces a novel approach to pipeline health monitoring by utilizing the unique strengths of a one-dimensional DenseNet model. By integrating the sequential data-handling capabilities of one-dimensional CNN with DenseNet’s efficient feature propagation, this model achieves a remarkable balance between computational efficiency and diagnostic precision. The DenseNet architecture ensures the reuse of learned features across layers, minimizing information loss and enabling the model to capture complex signal patterns without overfitting. This method not only enhances the accuracy of leak detection and leak size identification but also demonstrates robust performance against noise and variability in pipeline signals. By providing reliable insights into pipeline conditions, the proposed model reduces maintenance costs, prevents destructive failures, and ensures safer operations. This study bridges critical gaps in existing methodologies and establishes a scalable, real-time solution, marking a significant advancement in pipeline monitoring and strengthening the resilience of important infrastructure systems.

The key contributions of this study are as follows.

1. Development of a hybrid deep learning framework combining 1D-CNN and DenseNet for robust AE-based pipeline leak detection.
2. Implementation of an empirical wavelet transform with adaptive thresholding to optimize feature extraction and reduce signal noise interference.
3. Introduction of a novel multi-stage feature fusion approach, integrating both time-domain and frequency-domain AE features to enhance leak size classification accuracy.

This paper is organized as follows: Section 2 presents the proposed methodology. Section 3 provides the technical background relevant to this study. Section 4 details the experimental setup, and Section 5 explains the results and discussion. Section 6 concludes the study with key findings and insights.

## 2. Proposed Methodology

**Figure 1:**
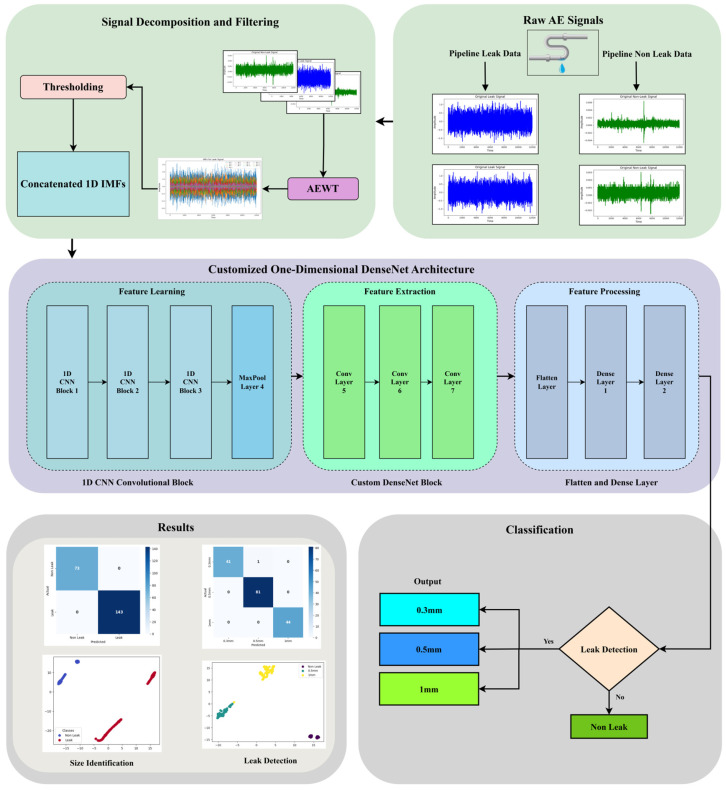
Graphical workflow of the proposed methodology.

This section presents a novel approach for pipeline leak detection and size identification using AE signals and deep learning techniques. The proposed methodology encompasses signal acquisition, processing, and analysis through a customized one-dimensional DenseNet architecture. Figure 1 illustrates the complete workflow of the proposed system.

Step I: AE signals are collected from the pipeline under leak and non-leak conditions. These signals contain valuable information regarding the pipeline’s structural condition.

Step II: The collected AE signals undergo preprocessing steps to ensure the data are informative for analysis. The process begins with the application of the EWT, which adaptively segments the signal into meaningful frequency bands. This segmentation is customized to extract the unique characteristics of each signal, allowing for the accurate isolation of features associated with each signal. Following this decomposition, adaptive thresholding is applied to refine the signal further by filtering out low-amplitude noise, effectively separating significant components from background interference. This step retains only the essential parts of the signal, preserving important information related to leak events. The denoised signals are then represented through a set of concatenated IMFs which hold useful features from the AE signal, providing comprehensive, noise-reduced representations.

Step III: After preprocessing, the AE signals are fed into a customized one-dimensional deep-learning model to extract features. This model operates in three stages to maximize feature extraction and processing efficiency. The process begins with a one-dimensional CNN layer that applies filters moving along the time dimension of the AE signal. These filters acquire temporal patterns indicative of potential leaks. Next, the model uses DenseNet layers arranged in a dense connectivity pattern, where each layer receives input from all previous layers. This configuration improves information flow through the model, ensuring that essential details are preserved and enhancing the model’s ability to detect minute, complex features within the signal. In the final stage, feature processing layers refine the extracted features further. The data are first flattened and then passed through dense layers that fine-tune the features, optimizing them for accurate leak detection and size classification in the final decision-making stage.

Step IV: The model undergoes thorough training and testing using real-world AE data collected from actual pipeline systems. The testing process verifies the model’s robustness, demonstrating its accuracy in detecting leaks and identifying leak sizes.

## 3. Technical Background

### 3.1. Acoustic Emission

Acoustic emission is the phenomenon where transient sound waves are generated within a material due to the sudden release of energy, typically resulting from structural changes such as cracking, deforming, or leaking. In pipeline systems, AE occurs when a leak releases elastic energy, which propagates as stress waves along the pipeline surface. AE sensors strategically placed along the pipeline detect these waves as transient signals called AE hits. These hits represent brief, high-energy events that capture important information about the pipeline’s structural condition and the characteristics of leaks. AE monitoring enables real-time detection of leaks by capturing and analyzing these transient signals. Since AE signals contain information about both normal and leak conditions, they must be processed to separate leak-related events from background noise. This is typically achieved by applying a threshold to distinguish real AEHs from operational noise. By extracting and analyzing key features of AE signals, such as peak amplitude, rising time, decay time, and average frequency, it is possible to gain insight into the severity and size of a leak, thereby supporting effective leak detection and size identification.

### 3.2. Adaptive Empirical Wavelet Transform

**Figure 2:**
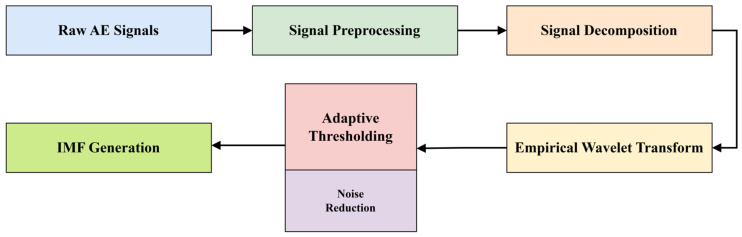
Flowchart of the signal preprocessing steps.

The overall flow of the adaptive empirical wavelet transform (AEWT) process is illustrated in Figure 2, providing a clear representation of the steps involved in separating leak-related AE features from operational noise.

**Figure 3:**
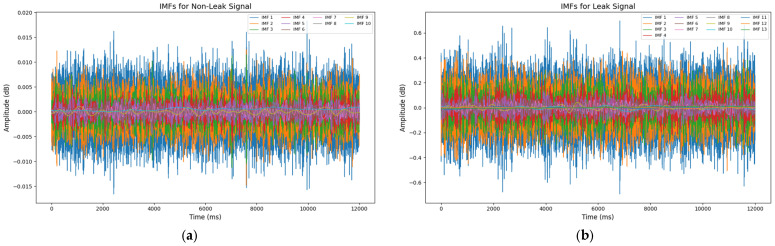
Intrinsic mode functions for (**a**) non-leak signal and (**b**) leak signal.

In this study, AEWT is used to split AE signals into distinct frequency components that are specifically associated with pipeline leaks. Unlike conventional wavelet transforms that use fixed wavelet bases, the adaptive method adapts its wavelet filters to align with the unique spectral characteristics of each AE signal, as shown in Figure 3. This adaptability makes it particularly effective for analyzing non-stationary signals, like those generated by pipeline leaks, as it allows the decomposition to focus on frequencies that reflect leak severity and other relevant features. First, the AE signal is transformed into the frequency domain using the Fourier Transform, which provides a frequency spectrum $X\left(f\right)$. This spectrum reveals the range of frequencies pin the AE signal, allowing it to adaptively select frequency bands based on local maxima corresponding to leak-related events. Mathematically, the Fourier Transform of the AE signal $x\left(t\right)$ is expressed in Equation (1) as

(1) $$X\left(f\right)=\int_{-\infty }^{\infty }x\left(t\right)e^{-2i\pi ft}\;dt\;$$

where $X\left(f\right)$ is the Fourier Transform of the time-domain signal, and $x\left(t\right)$ is the time-domain signal, representing a function of time. f is the frequency variable measured in Hertz (Hz). $e^{-2i\pi ft}$ is the complex exponential function that acts as a basis for decomposition.

AEWT then identifies key frequency bands by analyzing this spectrum, focusing on frequencies with local peaks that indicate potential leak-related features. For each identified frequency band, a unique wavelet filter $\psi_{i}(t)$ is constructed, where i represents the frequency range. This enables the decomposition of the AE signal. Mathematically, it is represented in Equation (2) as

(2) $$x\left(t\right)=\sum_{i=1}^{n}W_{i}(t)\psi_{i}(t)\;$$

In Equation (2), $W_{i}(t)$ denotes the wavelet coefficients for each band i, representing the energy within that band. Adaptively isolating these frequency components provides a refined signal structure that emphasizes features directly tied to leak events, preparing the data for subsequent noise reduction and feature extraction. This decomposition allows for the efficient separation of relevant leak frequencies from background noise, enhancing the ability to identify varying leak severities based on distinct frequency signatures.

#### Adaptive Thresholding

Adaptive thresholding is a dynamic technique that adjusts the threshold based on the local characteristics of each frequency band within the AE signals, making it particularly effective for isolating important leak-related signal components from noise. This technique is applied to each decomposed frequency band to further isolate leak-related signal components from background noise. Unlike static thresholding techniques, adaptive thresholding dynamically adjusts according to the statistical properties of each frequency band, making it more effective for identifying significant events in AE data. This approach calculates a unique threshold for each segment based on its local standard deviation, allowing for sensitivity to variations across frequency bands. The threshold for each segment is defined mathematically in Equation (3) as

(3) $$T_{i}=\;\alpha \cdot \sigma_{i}$$

In Equation (3), $T_{i}$ is the threshold for the $i^{th}$ frequency band, α is a scaling factor that controls sensitivity, and $\sigma_{i}$ is the standard deviation within the band. After calculating $T_{i}$, low-amplitude wavelet coefficients below than $T_{i}$ are filtered out, while those above the threshold are retained, ensuring that only the most meaningful components are kept for further processing. This can be represented mathematically in Equation (4) as

(4) $$W_{i}^{filtered}\;\left(t\right)=\;\left\{\begin{array}{c} W_{i\;}\left(t\right),\;\;\;\;\;\;if\;\left|W_{i\;}\left(t\right)\right|>T_{i\;}\\ 0\;,\;\;\;\;\;\;\;\;\;\;\;\;otherwise\;\;\;\;\;\;\;\;\;\;\; \end{array}\right.$$

where $W_{i}\left(t\right)$ is the original wavelet coefficient at scale i and time t. $W_{i}^{filtered}\left(t\right)$ is the filtered wavelet coefficient after applying the thresholding operation. $T_{i}$ is the threshold value for the wavelet coefficients at scale i. $\left|W_{i}\left(t\right)\right|$ is the magnitude of the wavelet coefficient. This filtering process retains frequency components directly linked to leak events, removing noise and unrelated data, thereby enhancing the signal’s relevance for leak detection and size identification. Following adaptive thresholding, the concatenated one-dimensional IMFs technique is used to unify the frequency components into a single, comprehensive representation. Each frequency band that remains after thresholding is converted into an IMF, capturing the distinct oscillatory mode of that band. The concatenation of these IMFs provides a consolidated view of the AE signal, ensuring that all relevant leak-related features are preserved in a single structure. The concatenated IMFs can be mathematically expressed in Equation (5) as

(5) $${IMF}_{conc}\left(t\right)=\;\sum_{i=1}^{n}{IMF}_{i}\left(t\right)\;$$

where ${IMF}_{i}(t)$ represents each individual mode derived from the adaptive thresholding process. To facilitate further processing, these concatenated IMFs are transformed into a frequency vector $V_{freq}$ via the Fourier transform, converting the time-domain signal into a frequency-domain representation as expressed in Equation (6).

(6) $$V_{freq}=F({IMF}_{conc}\left(t\right))$$

The frequency vector obtained from Equation (6) serves as the input to the subsequent deep learning model, providing a structured, noise-reduced dataset that highlights leak-specific information, thus enabling effective feature learning and classification.

### 3.3. Customized One-Dimensional DenseNet Architecture

The proposed pipeline leak detection and size identification system uses a customized deep learning architecture to analyze the behavior of AE signals. This architecture is specifically designed to capture, propagate, and refine features from AE signals, enabling accurate leak detection and size classification. The model consists of three main stages: a one-dimensional layers CNN for initial feature extraction, a DenseNet block for advanced feature propagation, and feature processing layers for final refinement and classification.

#### 3.3.1. One-Dimensional CNN

**Figure 4:**
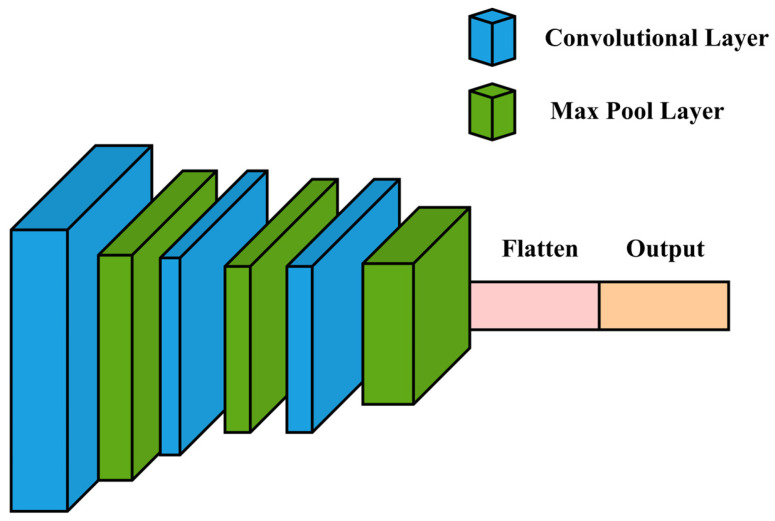
One-dimensional CNN architecture.

The initial stage of feature learning is implemented using a one-dimensional CNN block, which is well-suited for processing sequential data such as AE signals. The one-dimensional CNN architecture is composed of three sequential convolutional blocks, as shown in Figure 4. Each block applies filters that slide over the AE signal along the time dimension, capturing local patterns that indicate leak events. The convolutional operation for each layer can be represented mathematically in Equation (7) as

(7) $$y\left(t\right)=\sum_{k=1}^{K}w_{k}\cdot x\left(t+k\right)+b$$

In Equation (7), $y\left(t\right)$ is the output of the convolution at time t, $x\left(t+k\right)$ is the input signal, $w_{k}$ represents the weights of the filter of size K, and b is the bias term. This operation allows the model to capture shifts and fluctuations in the AE signal, correlating with leak events. The sliding filters in the 1D CNN layers detect temporal changes in the AE signal, such as amplitude and frequency fluctuations, that correlate with potential leak events. The model builds a hierarchy of features, from low-level patterns to more complex representations associated with leak signatures. After the third convolutional layer, a Max Pooling operation is applied to reduce the feature map’s dimensionality, as illustrated in Figure 4, and represented mathematically in Equation (8) as

(8) $$y_{pool}=\max\{y\left(t\right),\;y\left(t+1\right),\cdots ,y\left(t+p-1\right)\}$$

In Equation (8), p is the pooling size. This pooling step retains prominent features while reducing computational complexity, ensuring that important temporal relationships are preserved without overloading the model.

#### 3.3.2. DenseNet

**Figure 5:**
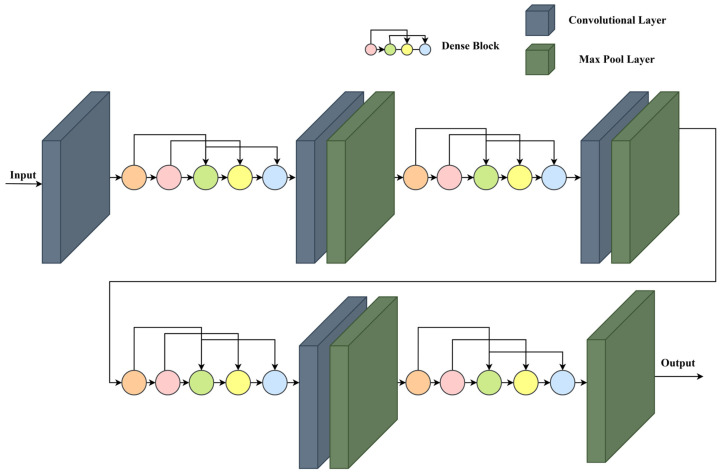
DenseNet architecture.

Following the initial one-dimensional CNN feature extraction, the model incorporates a DenseNet block to enhance feature propagation and ensure comprehensive learning of signal characteristics. As illustrated in Figure 5, the DenseNet block consists of three sequential one-dimensional convolutional layers followed by a MaxPooling1D operation to reduce the feature map’s dimensionality. The first convolutional layer, Conv1D (Dense Layer 1), employs 128 filters with a kernel size of 3 and processes an input of shape (Batch, 128, 6000), using the ReLU activation function to capture essential signal features. The second layer, Conv1D (Dense Layer 2), also utilizes 128 filters and the same kernel size, building upon the features extracted by the first layer. Similarly, the third layer, Conv1D (Dense Layer 3), refines and enhances feature representations by utilizing the outputs of the preceding layers. Finally, a MaxPooling1D layer reduces the time dimension by half, resulting in an output shape of (Batch, 128, 3000). This pooling operation ensures the retention of prominent features while minimizing computational complexity. This dense connectivity of the DenseNet block allows each layer to receive inputs from all preceding layers, promoting efficient feature reuse and ensuring the preservation of critical signal information. This architecture enhances gradient flow and reduces potential issues such as vanishing gradients, particularly in deeper networks. By capturing minute variations in frequency and intensity, the DenseNet block provides a robust framework for detecting and characterizing leaks of varying sizes in AE signals.

#### 3.3.3. Feature Processing

The final stage of the architecture focuses on processing and refining the features for accurate leak detection and size identification. After passing through the DenseNet block, the multi-dimensional feature map is flattened into a one-dimensional vector, simplifying the feature structure for the final classification layers. This relationship can be expressed as in Equation (9).

(9) flattened−vector=flatten(x)

The flattened vector obtained from Equation (9) is fed into two dense layers that fine-tune the feature representation.

(10) y=f(W·x+b)

In Equation (10), W is the weight matrix, b is the bias, and f is an activation function such as ReLU. These layers further refine the model’s understanding of the AE signal characteristics relevant to leak detection and size identification, optimizing the feature set for the classification task. As the data progresses through the dense layers, dimensionality reduction is applied to streamline the feature representation. This ensures that only the most relevant features are retained for classification, reducing computational load and enhancing classification accuracy.

In the final output layer, the model performs both leak detection (binary classification) and leak size identification (multi-class classification). The overall architecture of the one-dimensional DenseNet is mentioned in Table 1.

## 4. Experiment Setup

**Figure 6:**
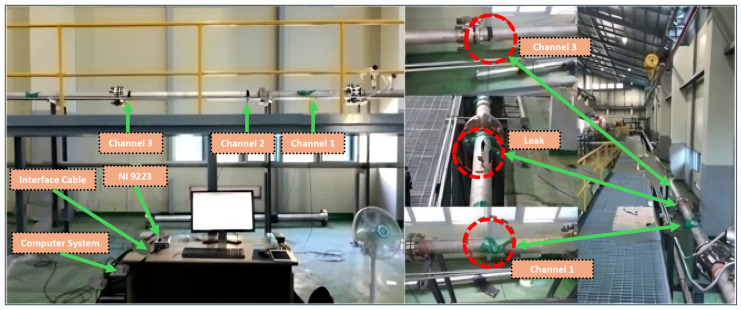
Experimental setup for pipeline leak detection.

**Figure 7:**
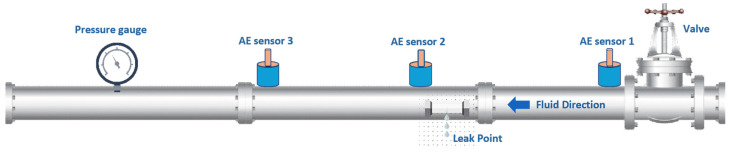
Pipeline architecture for the experiment.

The experimental setup for AE data acquisition utilized a stainless-steel pipeline with a 6 mm wall thickness and an outer diameter of 114 mm. Schematic representations of the setup are shown in Figure 6 and Figure 7. To simulate leaks of varying sizes, holes were drilled into the pipeline using an electric drill, and fluid control valves were welded near the holes to regulate the flow. Water and gas were used as the transporting fluids in the pipeline for data collection.

High-sensitivity R15I-AST sensors from MITRAS Corporation (Weiden in der Oberpfalz, Germany) were used to detect AE signals. These sensors were secured onto the pipeline using plastic tape, and a specialized coupling gel was applied to ensure optimal sensor-to-pipeline contact. The AE signals were captured using a National Instruments NI-9223 module (Austin, TX, USA) featuring a 16-bit analog-to-digital converter with adjustable sampling rates. Data acquisition occurred at a sampling frequency of 1 MHz, and the signals were stored on a computer with a 1-terabyte storage capacity. Before data collection, calibration of the AE sensors was conducted using the Hsu–Nelson method to verify the sensitivity and accuracy of the system. Once calibration confirmed optimal performance, the data acquisition process was started, with AE signals recorded under both normal and leak conditions. Detailed parameters of the R15I-AST sensors are presented in Table 2.

This study utilized datasets collected from a pipeline system operating under both normal and simulated leak scenarios. In normal conditions, the control valve was kept closed, maintaining fluid pressure via a centrifugal pump set to either 13 or 18 bar. Data were collected at a temperature of 25 °C. AE signals were recorded continuously over predefined intervals to capture uninterrupted data streams. Leak conditions were introduced by drilling circular openings of 0.3 mm, 0.5 mm, and 1 mm in diameter at both 13-bar and 18-bar pressure levels. The use of fixed hole diameters allowed for controlled and repeatable experiments, ensuring consistency in data collection. Since leak volume is influenced by multiple dynamic factors such as fluid pressure and flow conditions, defining leak size based on hole diameter provided a reliable and reproducible approach. The AE signals were continuously recorded under both normal and leak conditions, with data collected for two minutes in normal conditions and four minutes for each leak scenario. In total, 360 samples were obtained at each pressure setting: 120 representing normal conditions and 240 corresponding to various leak states. The details of the collected data are summarized in Table 3.

**Figure 8:**
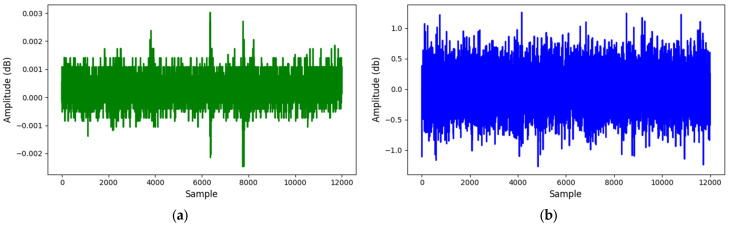
AE signals at 13-bar pressure: (**a**) normal; (**b**) leak.

**Figure 9:**
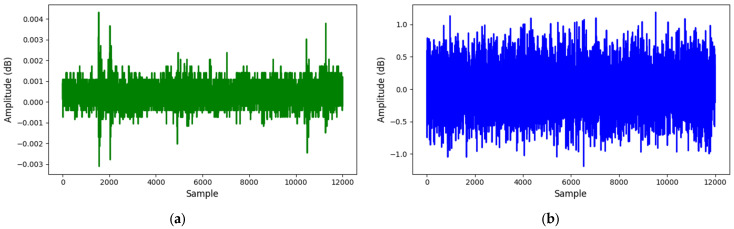
AE signals at 18-bar pressure: (**a**) normal; (**b**) leak.

Figure 8 compares AE signals between leak and normal states at 13 bar, while Figure 9 extends the comparison at 18-bar pressure. These visual representations highlight the differences in AE signals under normal and leak conditions, showing the reliability of the recorded data.

## 5. Result and Discussion

This study introduces a customized one-dimensional DenseNet architecture applied to AE signals for real-time leak detection and size identification in industrial pipelines. The proposed approach utilizes AE signal analysis and a customized model combining the strengths of one-dimensional CNN and DenseNet to capture both spatial and temporal features essential for the accurate detection and classification of pipeline leaks. The model’s performance was assessed across key metrics, including accuracy, precision, recall, and F1 score, to evaluate its performance in distinguishing between leak and non-leak conditions and identifying varying leak sizes.

The dataset used in this study includes 1080 AE signal samples collected under leak and non-leak conditions, with leak sizes of 0.3 mm, 0.5 mm, and 1 mm across fluid pressures of 13 and 18 bars. For training, 80% of the samples were selected randomly, while 20% were reserved for testing. To ensure reliability, experiments were repeated 15 times, providing a robust evaluation of model performance. During each iteration, the model was assessed on its ability to accurately detect leaks and classify leak sizes under varying pressure and size conditions. Metrics such as precision, accuracy, F1 score, and recall were used to evaluate the method’s performance in both detecting leaks and identifying their sizes. These metrics offer a clear measure of the classification algorithm’s effectiveness and accuracy in categorizing the data. The specific formulas used for these calculations are shown in Equations (11)–(14).

(11) $$Precision=\;\frac{{TP}_{\alpha }}{{TP}_{\alpha }+{FP}_{\alpha }}$$

(12) $$Recall=\;\frac{{TP}_{\alpha }}{{TP}_{\alpha }+{FN}_{\alpha }}$$

(13) $$F1\;Score=2*\frac{Precision*Recall}{Precision+Recall}$$

(14) $$Accuracy=\;\frac{{TP}_{\alpha }+{TN}_{\alpha }}{N}$$

In Equations (11)–(14), the terms ‘${FP}_{\alpha }$’, ‘${FN}_{\alpha }$’, ‘${TP}_{\alpha }$’, and ‘${TN}_{\alpha }$’ represent false positive, false negative, true positive, and true negative outcomes for class A, respectively. A false positive occurs when a sample is incorrectly assigned to class A, even though it does not belong there. A true positive indicates that samples genuinely from class A have been correctly identified. On the other hand, a false negative occurs when samples that belong to class A are incorrectly classified as not being part of it, and the true negative is that the samples were not placed in class A and did not belong to this class as well. The total number of samples in class A is denoted as N, which is the sum of ‘${FP}_{\alpha }$’, ‘${FN}_{\alpha }$’, ‘${TP}_{\alpha }$’, and ‘${TN}_{\alpha }$’.

### 5.1. Proposed Method

The proposed customized one-dimensional DenseNet model, applied to AE data from industrial fluid pipelines, demonstrates powerful capabilities in two important tasks: distinguishing between leak and non-leak conditions and accurately determining the size of detected leaks.

The model’s architecture combines the strengths of a one-dimensional CNN with the DenseNet framework, effectively addressing the complexities of AE signals. The one-dimensional CNN component excels in processing sequential AE data, enabling the model to isolate and extract important features relevant to both leak detection and leak size identification. This precise feature extraction is important for reliably identifying leak characteristics, contributing to accurate fault diagnosis. Meanwhile, the DenseNet framework carries efficient feature propagation through densely connected layers, ensuring the retention and reuse of important information as the data progresses through the network. This dense connectivity prevents the loss of essential details, enabling the model to distinguish between different leak sizes and conditions with high precision. By optimizing feature flow and enhancing the model’s capacity to recognize complex patterns within AE signals, the DenseNet architecture significantly improves overall model performance. The model’s performance, summarized in Table 4 and Table 5, is exemplary, achieving an accuracy of 99.76 for leak detection and 99.78% for leak size identification, respectively. Such high metrics indicate that the model performed better than the conventional methods in detection and classification accuracy, with its success largely due to the combined strengths of a one-dimensional CNN and the DenseNet framework.

### 5.2. Comparative Analysis

To thoroughly evaluate the performance of the proposed one-dimensional time series DenseNet model, we conducted a comparison against three established methods: a one-dimensional CNN model [49], a long short-term memory (LSTM) model [50], and the XGBoost model [26]. All models were tested using the same AE data under similar experimental conditions to ensure a fair comparison. This analysis allowed us to assess how effectively the proposed method performs in both areas, leak detection, and leak size identification, relative to these well-known techniques.

The first reference model, a one-dimensional CNN, follows the methodology proposed by Wang and Gao [49], who developed a pipeline leak detection method based on acoustic-pressure information fusion. This approach uses convolutional layers to extract spatial features from acoustic emission signals and pressure variations in pipelines. Their work demonstrated that combining these features improves the detection of leaks under various operating conditions. Similarly, in our experiments, the one-dimensional CNN processed the raw AE signals through multiple convolutional layers, achieving an accuracy of 98.31% for leak detection and 97.52% for leak size identification. While the model effectively captured spatial features to identify leaks, it struggled to discern minute temporal patterns required for accurate classification of leak sizes. This limitation is consistent with Wang and Gao’s findings, where spatial data fusion enhanced detection capabilities but fell short in capturing time-dependent leak characteristics. Consequently, the CNN’s performance declined in complex or noisy environments, making it less reliable for precise leak size differentiation.

The second reference model, an LSTM, was implemented based on the improved convolutional neural network approach by Xu et al. [50], which focuses on pipe leakage identification using acoustic emission data. Xu et al. proposed an LSTM-based framework capable of tracking the temporal evolution of acoustic signals to identify leaks accurately. This time-series modeling capability makes LSTM particularly effective for sequential data analysis, as highlighted in their study. In our experiments, LSTM achieved an accuracy of 96.13% for leak detection and 94.11% for leak size identification. While it excelled at recognizing time-dependent leak progression, the LSTM encountered limitations in spatial feature extraction, which Xu et al. also identified as a challenge for time-series models. The inability to capture detailed spatial patterns resulted in lower accuracy for leak size differentiation, especially in scenarios involving overlapping signal features. This limitation affected the model’s overall performance, particularly for smaller leaks or those occurring under complex operational conditions.

The third comparison model, an XGBoost machine learning approach, was implemented following the methodology outlined by Lee and Kim [26]. This model utilized vibration data from water pipelines, integrating various statistical and signal processing features for classification. XGBoost, a gradient-boosting algorithm, excels in structured data analysis and demonstrates competitive performance for leak detection. In our experiments, XGBoost achieved an accuracy of 95.24% for leak detection and 92.38% for leak size identification. Its reliance on pre-processed features and a robust classification framework enabled it to perform well under standard conditions. However, similar to the findings by Lee and Kim, this model struggled in environments with high noise levels leak signals, where feature generalization was critical. Additionally, the preprocessing and feature engineering requirements added complexity, making them less suitable for real-time applications compared to end-to-end deep learning models. Despite these limitations, its computational efficiency and strong classification performance make XGBoost a valuable baseline for comparison.

**Figure 10:**
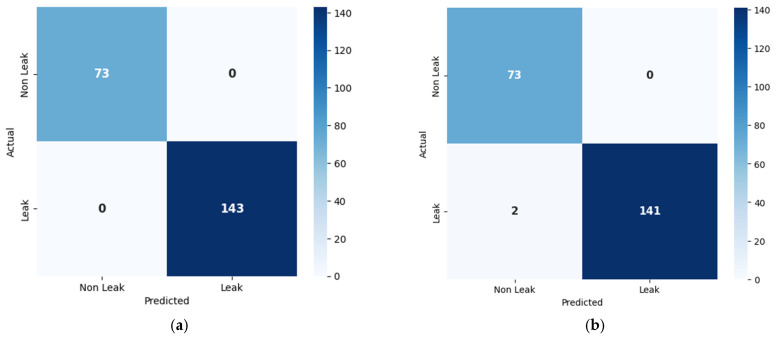

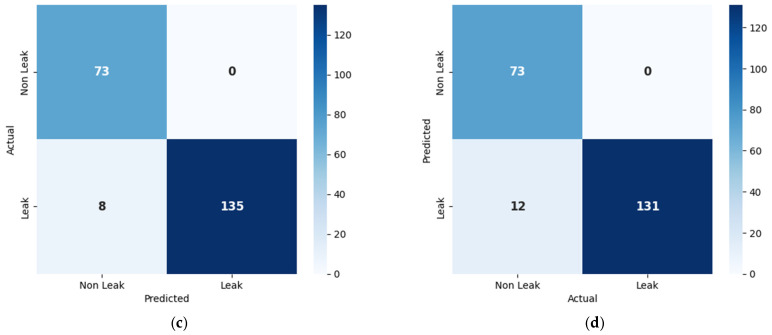
Confusion matrices for leak detection of (**a**) proposed method; (**b**) 1D CNN; (**c**) LSTM; and (**d**) XGBoost.

**Figure 11:**
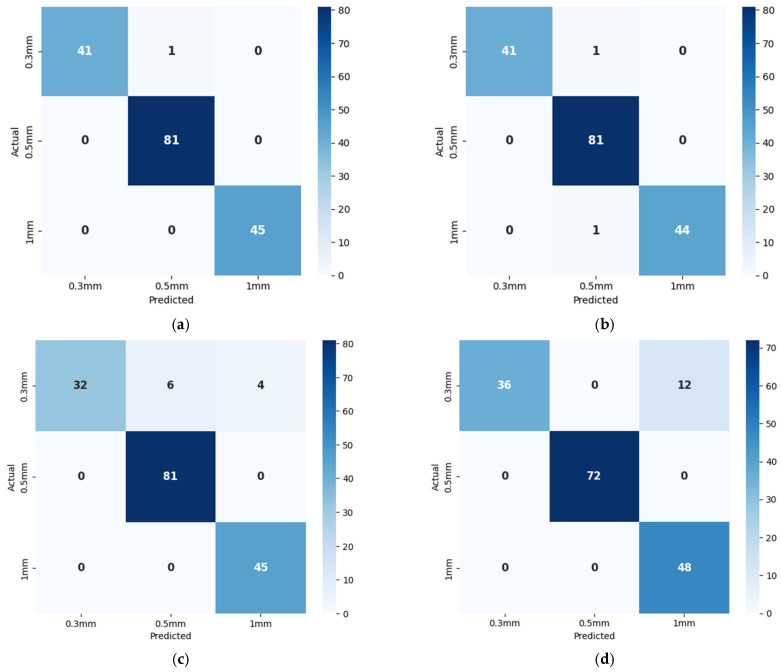
Confusion matrices for leak size identification of (**a**) proposed method; (**b**) 1D CNN; (**c**) LSTM; and (**d**) XGBoost.

**Figure 12:**
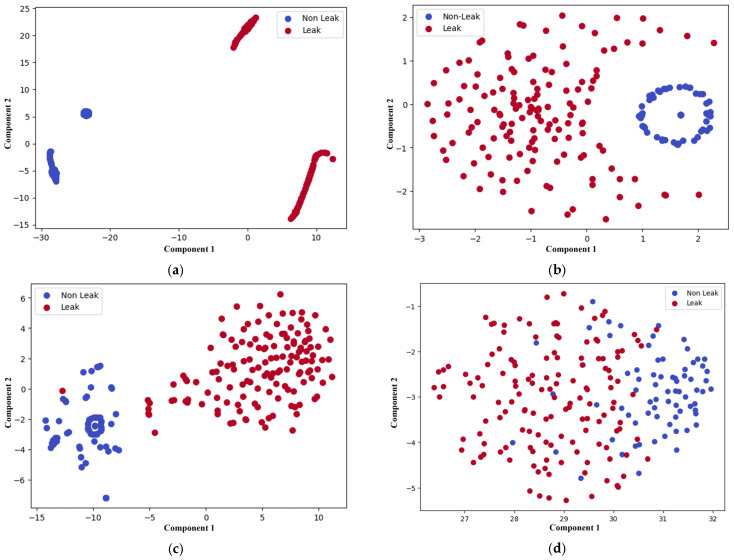
t-SNE plots for leak detection of (**a**) proposed method; (**b**) 1D CNN; (**c**) LSTM; and (**d**) XGBoost.

**Figure 13:**
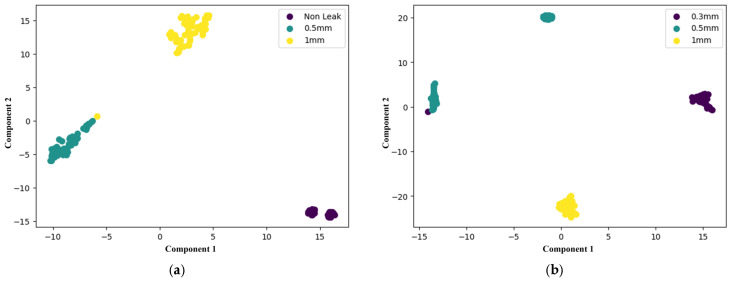

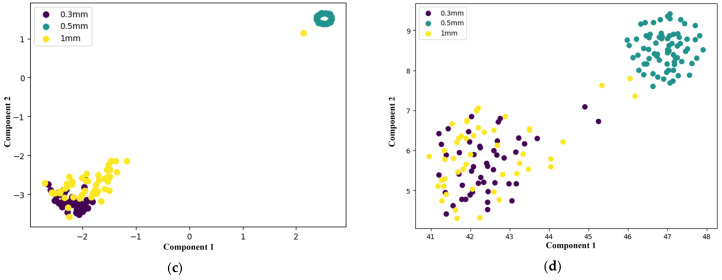
t-SNE plots for leak size identification of (**a**) proposed method; (**b**) 1D CNN; (**c**) LSTM; and (**d**) XGBoost.

The proposed method addresses the limitations of all three models by integrating spatial and temporal features more effectively through its DenseNet architecture, allowing it to detect leaks while accurately distinguishing between different leak sizes. This comprehensive feature extraction enables the model to perform better as compared to all the comparison models across all key metrics. While the one-dimensional CNN emphasizes spatial data, the LSTM specializes in temporal sequences, and XGBoost relies on structured features, the proposed model outperforms all by offering more robust and precise leak detection and classification capabilities. A confusion matrix is a table that summarizes the classification performance of a model by showing the number of true positives, true negatives, false positives, and false negatives. These values are used to compute accuracy, precision, recall, and F1 score. In this study, confusion matrices shown in Figure 10 and Figure 11 illustrate the classification performance of leak detection and leak size identification. The t-SNE (t-Distributed Stochastic Neighbor Embedding) visualization is a dimensionality reduction technique that helps to project high-dimensional data into a lower-dimensional space while preserving local structure. It is particularly useful for visualizing the clustering patterns of different classes. Figure 12 and Figure 13 present the t-SNE embeddings, highlighting how well the proposed method differentiates between leak and non-leak conditions. This strong, consistent performance across all metrics solidifies the proposed method as a powerful and effective tool for real-world pipeline monitoring, surpassing the individual capabilities of CNN, LSTM, and XGBoost models.

## 6. Conclusions

This study presents a novel AI-driven fault detection system utilizing a customized one-dimensional DenseNet model for real-time leak detection and size classification in energy pipeline networks. By using acoustic emission signals in conjunction with Empirical Wavelet Transform and adaptive noise thresholding, the system effectively isolates critical signal components indicative of leak events. DenseNet architecture excels in capturing temporal patterns and efficiently propagating features, enabling the precise detection and classification of leak sizes (0.3 mm, 0.5 mm, and 1 mm) with an average accuracy of 99.7%. The model’s robustness and scalability make it highly suitable for deployment in extensive pipeline systems, ensuring reliable performance under diverse operating conditions. The significance of this approach lies in its ability to address complex fault scenarios while minimizing computational overhead, facilitating its integration into pipeline integrity management systems. Furthermore, its adaptability to large-scale monitoring supports enhanced reliability, operational safety, and cost-effective predictive maintenance strategies in critical infrastructure systems transporting oil, gas, and other energy resources.

The study’s limitations include the dependency of the model’s performance on the quality of acoustic emission signals, which may decrease under extreme noise or interference, and the limited diversity of the training dataset, potentially affecting generalizability to unusual pipeline conditions. The experiment was conducted with a single leak location to ensure controlled conditions and consistent data collection. However, in practical pipeline systems, leaks may occur at multiple locations, which can affect AE signal characteristics. Future work will focus on expanding datasets to include various pipeline materials, environmental conditions, and operating pressures to enhance the model’s generalizability. Additionally, evaluating the model’s robustness across multiple leak locations will provide a more comprehensive understanding of its performance in real-world scenarios.

## Figures and Tables

**Table 1:** 1D-CNN and DenseNet model architecture.

Layer Type	Layer Name	Filters/Units	Kernel Size	Output Shape	Activation Function
Input Layer	Input	-	-	(Batch, 1, 12,000)	-
1D CNN Block	Conv1D (Block1)	16	3	(Batch, 16, 12,000)	ReLU
	Conv1D (Block2)	32	3	(Batch, 32, 12,000)	ReLU
	Conv1D (Block3)	64	3	(Batch, 64, 12,000)	ReLU
	MaxPooling1D (Block)	-	2	(Batch, 64, 12,000)	-
DenseNet Block	Conv1D (Dense Layer 1)	128	3	(Batch, 128, 6000)	ReLU
	Conv1D (Dense Layer 2)	128	3	(Batch, 128, 6000)	ReLU
	Conv1D (Dense Layer 3)	128	3	(Batch, 128, 6000)	ReLU
	MaxPooling1D (Dense Block)	-	2	(Batch, 128, 3000)	-
Feature Processing	Flatten	-	-	(Batch, 384,000)	-
	Dense (Fully Connected 1)	128	-	(Batch, 128)	ReLU
	Dense (Fully Connected 2)	64	-	(Batch, 64)	ReLU
Output Layer	Dense (Leak Detection)	1	-	(Batch, 1)	Sigmoid
	Dense (Leak Size Output)	3	-	(Batch, 3)	Softmax

**Table 2:** Detail parameters of the R15I-AST sensor.

No.	Elements	Value
1	Maximum sensitivity, ref [V/(m/s)]	109 [dB]
2	Maximum sensitivity, ref [V/µbar]	22 [dB]
3	Range of operating frequency	50–400 [kHz]
4	Resonant frequency, ref [V/(m/s)]	75 [kHz]
5	Resonant frequency, ref [V/mbar]	150 [kHz]
6	Directionality	±1.5 [dB]
7	Temperature range	35 to 75 [°C]
8	Thickness of pipeline	6.02 mm
9	Material of pipeline	304 stainless steels
10	The outer diameter of the pipeline	114.3 mm

**Table 3:** Details of the data collected during the experiment.

Dataset	Pressure of Fluid (Bars)	Leak Size (mm)	Time (s)	Number of Samples	
				Non-Leak (Normal)	Leak
Water	13	1.0	360	120	240
Water	18	0.5	360	120	240
Gas	13	1.0	360	120	240
Gas	18	0.3	360	120	240

**Table 4:** Comparative result of leak detection.

Models	Accuracy (%)	Precision (%)	Recall (%)	F1 Score (%)
Proposed	99.76	99.32	99.63	99.47
1D CNN [49]	98.31	98.43	98.13	98.28
LSTM [50]	96.13	95.37	97.12	96.24
XGBoost [26]	95.24	94.38	96.73	95.54

**Table 5:** Comparative result of leak size identification.

Models	Accuracy (%)	Precision (%)	Recall (%)	F1 Score (%)
Proposed	99.78	99.31	99.32	99.31
1D CNN [49]	97.52	98.12	97.53	97.82
LSTM [50]	94.11	94.34	92.53	93.43
XGBoost [26]	92.38	93.21	92.52	92.86

## Data Availability

Data are contained within the article.

## Nomenclature

AE	Acoustic emission
EWT	Empirical Wavelet Transform
CNN	Convolutional Neural Network
AEWT	Adaptive Empirical Wavelet Transform
IMF	Intrinsic Mode Function
TD	Time Domain
FD	Frequency Domain
TFD	Time-Frequency Domain
STFT	Short-time Fourier transform
FFT	Fast Fourier transform
DL	Deep learning
ReLU	Rectified neural network
